# Improving the production of applied health research findings: insights from a qualitative study of operational research

**DOI:** 10.1186/s13012-017-0643-3

**Published:** 2017-09-08

**Authors:** Sonya Crowe, Simon Turner, Martin Utley, Naomi J. Fulop

**Affiliations:** 1 Clinical Operational Research Unit, University College London, 4 Taviton Street, London, WC1H 0BT UK; 2 Department of Applied Health Research, University College London, 1-19 Torrington Place, London, WC1E 7HB UK

**Keywords:** Knowledge production, Operational research, Translational health research, Auto-ethnography

## Abstract

**Background:**

Knowledge produced through applied health research is often of a form not readily accessible to or actionable by policymakers and practitioners, which hinders its implementation. Our aim was to identify research activities that can support the production of knowledge tailored to inform policy and practice. To do this, we studied an operational research approach to improving the production of applied health research findings.

**Methods:**

A 2-year qualitative study was conducted of the operational research contribution to a multidisciplinary applied health research project that was successful in rapidly informing national policy. Semi-structured interviews (*n* = 20) were conducted with all members of the project’s research team and advisory group (patient and health professional representatives and academics). These were augmented by participant (> 150 h) and non-participant (> 15 h) observations focusing on the process and experience of attempting to support knowledge production. Data were analysed thematically using QSR NVivo software.

**Results:**

Operational research performed a knowledge mediation role shaped by a problem-focused approach and an intent to perform those tasks necessary to producing readily implementable knowledge but outwith the remit of other disciplinary strands of the project. Three characteristics of the role were found to support this: engaging and incorporating different perspectives to improve services by capturing a range of health professional and patient views alongside quantitative and qualitative research evidence; rendering data meaningful by creating and presenting evidence in forms that are accessible to and engage different audiences, enabling them to make sense of it for practical use; and maintaining perceived objectivity and rigour by establishing credibility, perceived neutrality and confidence in the robustness of the research in order to unite diverse professionals in thinking creatively about system-wide service improvement.

**Conclusions:**

Our study contributes useful empirical insights about knowledge mediation activities within multidisciplinary applied health research projects that support the generation of accessible, practice-relevant and actionable knowledge. Incorporating such activities, or a dedicated role, for mediating knowledge production within such projects could help to enhance the uptake of research findings into routine healthcare and warrants further consideration.

**Electronic supplementary material:**

The online version of this article (10.1186/s13012-017-0643-3) contains supplementary material, which is available to authorized users.

## Background

In response to the complex, multi-faceted nature of service improvement in healthcare, applied health research increasingly uses multidisciplinary approaches to generate knowledge about different aspects of a problem and to give deeper insights than those available from each discipline alone [1, 2]. However, the knowledge thus produced is often of a form that is not readily accessible to, or actionable by, policymakers and practitioners, which can hinder its implementation into routine healthcare [3, 4]. There is growing recognition that implementation science must address methods not only for translating research findings in the literature into routine practice, but also for producing research findings that are more readily implementable in the first place [5].

For example, it has been proposed that research would be more readily implemented if the eventual users of its findings are involved in the research process rather than researchers seeking to mobilise knowledge to its intended audience only at the end of projects, if at all [4, 6–8]. Increasingly, multidisciplinary applied health research projects include patient and health professional representatives alongside academics from quantitative and qualitative disciplines. However, such projects can be challenging to operationalise and do not necessarily reap the full benefit of multi-stakeholder involvement [9]. For example, organisational, professional, social and epistemological boundaries can impact on knowledge sharing between those involved [10–12], with the productiveness of discussions about service improvement potentially limited by the dynamics between different health professional groups [13] and between practitioners and academics [14]. In particular, difficulties appreciating the quality and meaning of discipline-specific research outside one’s own area of expertise can hinder the production of research that relies on interaction among researchers and practitioners with different disciplinary backgrounds.

Proposed strategies for bridging the perceived boundaries between academics, practitioners and service users tend to be framed around the challenge of mobilising or implementing research findings in practice. For example, the role of “boundary spanners” in mobilising research findings has been explored in the literature both in terms of (human) knowledge brokers and (material) boundary objects [15]. In contrast, there is a paucity of empirical research on how knowledge is shared, integrated and created by diverse groups of professionals within applied health research projects and how knowledge production in such contexts can be tailored to policy and practice in addition to generating research publications [9, 16]. Bowen and Graham have argued [5] that, in order to promote research relevance and utilisation, it may be helpful to shift from the perspective of knowledge translation to that of effective knowledge production through “engaged scholarship” [17]. This is based on the belief that more relevant research comes from solution-focused collaborative inquiry that leverages the different perspectives of academics, practitioners and service users to generate useful knowledge [5, 17, 18].

Operational research (OR) can be defined as the “discipline of using models, either quantitative or qualitative, to aid decision-making in complex implementation problems” [19] (see Table 1). In a recent case study of an OR project that informed changes to a stroke pathway [20], Heaton et al. [21] liken the collaborative style of OR to engaged scholarship and identify this as a characteristic of successful co-production of knowledge for practice. Monks [19] conceptualises OR as an approach grounded in upfront systems thinking before action to alter a service is taken and describes three roles for OR within implementation science: OR to structure an implementation problem, OR as a tool for prospective evaluation and OR as a tool for strategic reconfiguration. Our work builds on this by demonstrating and characterising an additional role for OR as a mediator of knowledge production in applied health research projects. In doing so, it contributes useful empirical insights about activities within a research project that can support the production of knowledge tailored to informing policy and practice and so, ultimately, enhance research uptake.

**Table 1:** Operational research (OR)

Operational research (OR) is the discipline of using models and analysis to aid decision-making in complex systems and has been used in healthcare since the 1950s [19]. The features of the problem being addressed inform the choice of approach adopted by OR practitioners, which can include qualitative methods such as problem structuring and conceptual modelling as well as quantitative data analysis, mathematical modelling and simulation techniques [46].	

In this paper, we report a qualitative study of the contribution of OR to a United Kingdom (UK) grant-funded multidisciplinary applied health research project that was successful in rapidly informing national policy (see Table 2 for details) [22, 23]. OR was the approach deployed within the project to ensure the production of knowledge that could directly inform policy and practice, so we sought to address the research questions:

1. What were the characteristics of the OR approach that ensured the successful production of rapidly implementable knowledge?
2. What were the challenges of performing the OR role, and how were these overcome?
3. What insights can be drawn from this example about the skills and activities within research projects that could support the production of effective applied health research?

**Table 2:** How operational research was deployed within the applied health research project

An operational researcher (lead author of this article, SC) supported knowledge production within a 2-year multidisciplinary grant-funded research project focusing on services following discharge from infant cardiac surgery [22]. That research project had two objectives: firstly, to understand the challenges encountered in accessing and providing services and to identify patient risk factors for adverse outcomes through qualitative and quantitative strands of research, and secondly, for an advisory group to develop recommendations for improving services. The operational researcher joined the project in order to bridge these two objectives by providing an explicit process for translating the research findings into practical recommendations of relevance to policymakers. A combination of problem structuring (soft systems methodology [47]) and quantitative OR methods (classification and regression tree (CART) analysis [48] and data visualisation) were deployed. The technical details are described elsewhere [45, 49], but for the purposes of this article, we highlight four key aspects: - Developing a Rich Picture (a device used in soft systems methodology) to explore the key features of services following infant cardiac surgery, perceived problems and possible improvements (see Additional file 1) [45]. - Facilitating a workshop with the project’s advisory group, who were tasked with agreeing a set of recommendations for service improvement [45]. This included a patient representative from the charitable sector and representatives from tertiary, secondary, primary and community care. - Creating a visual representation of data analysis to inform the advisory group’s consideration of the prioritisation of service improvements (the CART diagram, see Additional file 2) [45]. - Using a systematic process to integrate findings from the different strands of research and recommendations from the advisory group and a parent workshop (using a hyper-framework [49]). Outputs from the applied health research project included published academic articles reporting findings from the quantitative [50] and qualitative [51–54] strands of research, as well as the final evidence-informed recommendations for service improvement [49]. These recommendations fed into National Health Service (NHS) England’s national review and public consultation on the care standards and specifications for commissioning specialist services for congenital heart disease [23]. Given the rapid uptake of the research outputs by national policymakers, we considered this to be a successful example of producing readily implementable knowledge within an applied health research context. We therefore sought to identify aspects of the OR approach to knowledge production that contributed to this success.	

## Methods

### Study design

Our 2-year study of the role of OR in a UK grant-funded multidisciplinary applied health research project (Table 2) had three components: (1) semi-structured interviews with those involved in the applied health research project, focusing on the characteristics and impact of the OR role; (2) participant observations by the operational researcher (lead author of this article, SC), focusing on their experience of performing the role, including the challenges it presented and how some of these were overcome; and (3) non-participant observations of key project meetings and advisory group workshops by a social scientist (ST) focusing on the contribution of OR to knowledge production. The study had Research Ethics Committee approval and all interviewees provided informed consent.

### Data collection

Two phases of semi-structured interviews were conducted (total *n* = 20). The first was with the research project team whilst the project was ongoing (*n* = 5). The second was following completion of the project with members of the project team (*n* = 4; *n* = 3 of whom were also interviewed in the earlier phase), participants in the project advisory group (*n* = 8) and recipients of the project findings at NHS England (*n* = 3). Interviews with the project advisory group included a mixture of junior and senior health professionals from community (*n* = 2), primary (*n* = 1), secondary (*n* = 1) and tertiary (*n* = 3) care settings and a patient representative (*n* = 1). Our sampling strategy was non-selective in that all members of the research project team and advisory group were interviewed. SC conducted all interviews, the first *n* = 3 of which were jointly conducted with ST. Interviews lasted between 28 and 87 min (mean 57 min) and were recorded and transcribed verbatim. Participant observations of formal and informal interactions were conducted throughout the research project by the operational researcher (SC; *n* > 150 h). Non-participant observations of key project meetings and advisory group workshops (ST; *n* > 15 h) supported the conduct and analysis of the interviews.

### Researcher reflexivity

In qualitative enquiry, the researchers are the principal research tool [24] and their relationship with participants and the extent of their interactions can effect participants’ responses as well as the researchers’ understanding of the phenomena [25]. Researchers therefore need to reflect critically about the implications of their positionality, which was particularly important for us given that the lead author (SC), who conducted interviews and participant observations, was also the operational researcher in the applied health project being studied. This meant they benefited from familiarity with the topic area gained through participation but needed to maintain analytical distance whilst being close to and invested in the project. In an attempt to ensure sufficient critical distance in the data collection and analysis, SC held frequent reflective debrief meetings on the planning, collection and analysis of data with a non-participant researcher (ST), who also carried out non-participant observations and jointly conducted some of the interviews. Two additional members of the analysis team who were not involved in data collection (MU, NJF) provided further critical distance. For further discussion on the potential implications of our study design, see the “Strengths and limitations” section.

### Data analysis

Interview data were coded using QSR NVivo (software designed to help organise and analyse qualitative data) and reviewed at regular debrief meetings by SC and ST who analysed them thematically, initially using an inductive approach that focused on the emergent characteristics of OR that supported knowledge production. All authors read the first phase of transcripts and agreed on a preliminary set of thematic categories based on the inductive analysis and drawing on the wider knowledge production literature. This provided a framework that informed the second phase of data collection and against which all transcripts were then analysed. Two researchers (SC, ST) independently tested and refined the initial framework through seeking negative cases and divergent data across all transcripts, reorganising and collapsing the data into overarching themes which were then agreed through discussion with all authors.

## Results

### Interviews: the characteristics of OR as a mediator of knowledge production

In general, participants in the applied health research project felt it had been successful in generating research directly relevant to improving services and attributed this in part to the contribution of operational research:

One of the things that frustrates me the most about pieces of research is that they’re not leading you to improve services and I felt that that was one of the differences with this piece of research and that was clearly one of your [operational researcher] remits, which was heartening. [Patient representative, project advisory group]

However, as captured by one participant in particular, they generally felt “a bit unclear about the boundaries of operational research” and that it involved “applying a range of skills to a particular problem but in a varied way” and was not “just thinking about particular methodologies, but actually thinking about that broad sense of that translation” [clinical research fellow, project study team]. The role OR performed in contributing to knowledge translation was likened to “having a mediator” [cardiologist, project study team].

Three overarching themes relating to the characteristics of OR as a mediator of knowledge production emerged from the interviews: (1) engaging and incorporating different perspectives to improve services (capturing a range of health professional and patient views alongside quantitative and qualitative research evidence), (2) rendering data meaningful (creating and presenting evidence in forms that are accessible to and engage different audiences, enabling them to make sense of it for practical use), and (3) maintaining perceived objectivity and rigour (establishing credibility, perceived neutrality and confidence in the robustness of the research in order to unite diverse professionals in thinking creatively about system-wide service improvement). We discuss these themes in detail below.

#### Engaging and incorporating different perspectives

The relevance of research and evidence to service improvement was considered to have been enhanced by engaging and incorporating different service and user perspectives alongside a variety of quantitative and qualitative data:

my sentiment on the evidence was, yes, you’ve drawn on it but some of the recommendations and conclusions of the study have come from talking to clinicians working on the ground about the practicalities of delivering care. [Secondary care paediatrician, project advisory group]

Both social and material aspects of the OR approach helped to enable appreciation of diverse forms of knowledge. For instance, some clinicians may be unreceptive to qualitative research evidence, suggesting a potential role for OR methods to present data in ways that would appeal to them:

health professionals are very, I think, often suspicious about qualitative information, unless it is presented to them in the right way. Like, particularly, kind of, cardiology, cardiac surgery. It is a very unfamiliar thing to them. They don’t know very much about it, and I think they respond quite well to some of the, kind of, more structured-, I don’t know, some of the ways that concepts are framed. By the things I’ve seen in the past from their OR team I think it helps them to, kind of, take stuff in. [Consultant intensivist, project study team].

Participants identified a facilitated workshop that was part of the OR problem structuring methodology as particularly important in enabling a broad range of representatives to contribute their practical knowledge of delivering care. They valued this rare opportunity to learn about the experiences of families and health professionals in different parts of the system. Meeting in person helped participants to appreciate the difficulties others were facing, which informed their discussions about how services could be improved.

it was very helpful to have those people [in the workshop], because as a specialist centre worker, you don’t necessarily appreciate some of the stresses and difficulties that the secondary and particularly primary care people have. [Consultant paediatric cardiologist, project advisory group]

The non-participant observations highlighted the importance of “soft skills” used by the operational researcher in leading the project groups’ translation of research findings into recommendations for policy and practice (see Table 4). The production of material artefacts to capture diverse stakeholder perspectives and integrate these within emerging project findings prior to the workshop was also reported as helpful. This included seeking participants’ views through group and one-to-one meetings and incorporating these in a visual, system-wide depiction of professional and patient narratives called a “Rich Picture” (see Table 2 and Additional file 1). Participants identified their own experiences in the picture and were able to situate these within the context of other services and the care journey families experienced, which they found both useful and interesting. The Rich Picture supported meaningful interactions on the day as participants were primed and willing to listen to others and consider improvements in different parts of the system from multiple perspectives:

People saw immediately what it [Rich Picture] was doing and knew which corner of the world that they were in. If I live here I could see that this was only a small section of the territory. Even if you’re just working in improving things here, it absolutely demands that you at least recognise that these other places exist. [Cardiologist, project study team]

More broadly, OR was considered to have played a key role in integrating the perspectives of the eventual knowledge users in the production of the research outputs and helping people from different communities to work together in considering improvement. As one participant reflected, OR “formed a bridge between the people who are experts at numbers, and the people who are working on the ground in the community, in order to make some of the processes a little bit more transparent” [General practitioner, project advisory group].

#### Rendering data meaningful to people and for improvement

The productiveness of the advisory group’s interactions about service improvement was enhanced by activities to render data generated in the project meaningful to them, both as individuals and as a group, and relevant to their task of developing recommendations. For example, interviewees reported that data analysis had been designed and presented in a manner that was accessible to participants across different disciplinary or professional backgrounds. Specifically, the presentation of complex quantitative information in a graphical format (the “CART diagram”, see Table 2 and Additional file 2) that participants found unintimidating and straightforward to interpret enabled participants to understand, and be confident of understanding, the relevant evidence sufficiently to draw on it in the workshop. They felt that it was a communication and sense-making tool that prompted and empowered people to question the quantitative research findings and stimulated discussions about service improvement:

If you’re not mathematically minded you can still understand it [CART diagram]. I think this really helps people not to feel frightened or to feel that they’re not getting it and, actually, then they can really think about what it means […] I think they teased it apart rather than just accepted it at face value […] as a communication tool as well, to get people thinking, talking, discussing in relation to, “Well, I actually want to think about the intervention, who are we going to be applying it to?” [Health psychologist, project study team]

The quantitative information and graphical output were shared with all members of the project advisory group prior to the facilitated workshop, and one-to-one meetings were held with those that had not been involved in earlier stages of the project in order to explain emerging research findings and preparatory material for the workshop, which they reflected enabled them to contribute more effectively:

I had a huge advantage in that you’d [operational researcher] come and seen me and we’d gone through a lot of this on a one-to-one basis, which was fabulous, I did find that very useful. Obviously I was being introduced to you but I was also being introduced to the way that you were going to present things. [Patient representative, project advisory group]

The variety of formats used to present emerging project findings (including written, graphical and pictorial) helped to engage participants from diverse professional backgrounds and according to personal preferences. For example, whilst nurses in particular found the Rich Picture engaging with its speech bubbles articulating familiar conversations they have with families, some participants (including the specialist doctors and patient representative) were more interested to see the graphical representation of quantitative data (the CART diagram). Many participants found these two research artefacts complementary in exploring different aspects of the problem and felt that they had helped to “facilitate discussions” (clinical research fellow, project study team) between people from different communities and enhanced the quality of stakeholder interactions about improvement.

#### Maintaining perceived objectivity and rigour

There was a common perception that the data and views collected in the project had been considered fairly and systematically and that the comprehensive and objective approach used when integrating findings from different sources to generate the project’s outputs (the “hyper-framework”, see Table 2) had enhanced their quality.

it [OR] has made it [the project’s recommendations] more comprehensive and it’s made it a bit more objective as well, because the problem is that whoever writes it, will bring to bear their own biases or their own perspective. So, if you have a way of, sort of, stepping back and being more objective and systematic, then you can, kind of, remove some of the human frailties that make something less good quality at the end. [Consultant intensivist, project study team]

Discussions in person and by email gave participants multiple opportunities to ask questions and helped to build their trust in the research processes and data, and to establish the credibility of the operational researcher in their mediating role. In the same way that participants valued the objectivity of the research processes, they considered it helpful that the operational researcher was independent and neutral. By virtue of being non-clinical and not strongly associated with any one aspect of the study, they were considered to be freer from clinical or disciplinary bias, professional hierarchy, affiliation to a particular organisation and entrenched views about how existing services and professional groups work (although the non-participant observations suggested that negotiating the operational researcher’s role does involve a political aspect, as described in Table 4). The operational researcher’s role was thought to be a privileged position that enabled a refreshing and creative way of considering service improvement:

It’s [OR] been refreshing and a different way of looking at things and free from the restrictions of […] a professional hierarchy or necessarily other working relationships where other factors come into play about conclusions you might want to come to or things you might want to say. I think you [operational researcher] felt like part of the team but an independent part of the team who has been unbiased. [Secondary care paediatrician, project advisory group]

The perceived independence of the operational researcher was also thought to have permitted them to ask what some might consider “naïve” questions that health professionals might not think to ask or want to be seen to ask, and to constructively challenge the underpinning rationales behind current and potential service provision in a way that strengthened the project output. Participants felt that the operational researcher probed certain aspects of the research process, such as how disparate forms of evidence collected in the study were being used to inform service improvement, prompting the project team to reflect on and refine their approach.

I think you’ve [operational researcher] been a very good, sort of, questioning presence in some of those meetings which has got us to sort of reflect on our process a bit more than I think we otherwise would have done. [Clinical research psychologist, project study team]

Interviewees reflected that the operational researcher demonstrated depth of understanding in each area of the project whilst retaining a clear overview of how different strands of the project informed each other, which they felt helped to unite people from diverse professions and disciplines around the project remit.

I don’t think other people had that same perspective as you [operational researcher] had and I don’t think there was anyone actually who was going to be necessarily willing or able to get that in depth overview. […] In depth of all of the different bits, and to be able to pull it all together in that way and I think you kind of kept everybody in there. [Health psychologist, project study team]

As one participant reflected, “once somebody has got the clarity, if they’ve really got it, everybody will agree with it and then it will seem trivial that it’s actually emerged as an issue at all” [Cardiologist, project study team].

Finally, effective communication emerged as important to all three themes, in particular being “good at listening and taking on board what other people say” [Consultant intensivist, project study team], and demonstrably integrating their views into the processes and outputs of the project. The ability and willingness to articulate project findings and raise awareness of the work externally, for example through the consultation process for a national review of services for congenital heart disease, was also seen as important to the wider application of the knowledge generated in the project.

I think you [operational researcher] did a very good job of advocacy for your work in raising our awareness of it and making sure that we knew it was going on and what exactly was going on, and making sure everybody was clear what the findings were. [Member of NHS England review team]

### Participant observations: performing the role of a mediator of knowledge production

Table 3 presents an account, based on field notes from participant observations, of what performing the knowledge mediation role involved in practice for the operational researcher, including some of the challenges it presented. Their role was a late addition to the research project so was shaped by the existing research objectives, methodological strands and other features of the project and research team. This flexibility of role definition is fairly typical for OR, where the nature of the problem and the requirement to work to the priorities of decision-makers inform the approach taken by the practitioner (see Table 2). This may involve, as it did in this project, drawing on methods in which the operational researcher has varying degrees of existing expertise, as well as allocating time and priority to the tasks needed for the project, sometimes at the expense of personal comfort or interest.

**Table 3:** An account, based on field notes from participant observations, of mediating knowledge production in an applied health research project

I was not involved in the grant proposal for the research project and came to know about it later on through the principal investigator, who I had collaborated with before. There appeared to be opportunities for OR to add value, so I joined the research team a few months into the project using separate funding from a personal fellowship. Members of the team were already assigned to particular strands of research (e.g. a clinical research psychologist was conducting staff and family interviews in the qualitative strand). As a free resource without a specified role in the grant proposal, I was flexible to use OR however might serve the project’s aims, primarily in relation to developing recommendations for service improvements on the basis of the evidence the study planned to collect: *In my view, there was no explicit method for doing this part of the project (drawing together strands and developing recommendations) and so we’re flexible to (and need to!) design that now*. [Participant observation field notes, February 2014] I worked collaboratively with the rest of the project team, across all research strands, with my approach informed by their ideas and sensitive to the need to keep people on board. For example, in one project meeting, I discussed my early thoughts on how to bring together findings from the different strands systematically and illustrated to the team how this might be done using a grid-like framework. This received a mixed response, from very positive to very negative, prompting a detailed discussion and, eventually, agreement about how to augment my idea: *It certainly stimulated debate! And debate that has led us to a better place in terms of understanding and documenting our process in a manner in which we are all happy with […] it seems important that the idea that this is useful for the project has been reached by the research team as a whole. This way it is a constructive thing that everyone wants to be done and sees the value in - and how their work fits into it*. [Participant observation field notes, March 2014] Method selection was not solely a technical decision and also involved considering the context and requirements of the project alongside my expertise. For example, a large and unanticipated part of my contribution was developing an analysis dataset from two national audits because I had relevant skills and prior experience that others in the team did not have (“the skillset needed to, kind of, bring the data set to order I think is something that only you were able to do in the project” [consultant intensivist, project study team]). This seemed of benefit to the project but was time consuming and frustrated me when it delayed my progress in other areas: *I had a growing sense of frustration mixed with panic during this meeting as I realised how much there still is to do - and that a lot of this falls down to my responsibility. They [the project team as a whole] have hugely underestimated how much work this dataset preparation and linking involves. This is going to take quite a bit more of my time and I want to be cracking on with the other OR side of things*. [Participant observation field notes, October 2013] I was already proficient in data analysis of this kind but less experienced in another technique I wanted to use, soft systems methodology (SSM), so my confidence to push, and ability to conduct, different aspects of the OR approach varied considerably: *Much of the OR part of the project is unknown territory for me, in that I am not that familiar with the techniques of SSM - so I feel some reticence to push forward with that, which means I am susceptible to focusing on this data analysis which is much more in my comfort zone*. [Participant observation field notes, November 2013] It took time to gain expertise in the areas I was less familiar with. Indeed, I found translating the evidence into recommendations for improvement very time consuming because of the scale and breadth of tasks it required and lack of significant dedicated resource for this purpose: *Even from the point of post-workshop, pulling together the final recommendations for endorsement took a lot of work! And a lot of my time […] whilst the team thinks it*’*s really important to have something coming out of the study in terms of practical implementation, they don*’*t have any time for it...* [Participant observation field notes, November 2014]	

### Non-participant observations: biscuits and politics

Table 4 presents an account, based on non-participant observations, of knowledge production during project meetings and the perceived influence of the operational researcher’s role in this process. The non-participant researcher’s perspective confirmed the importance of material devices (e.g. visual artefacts) in supporting the project group’s decision-making and the operational researcher’s role in helping to draw out the perspectives of different groups (e.g. by asking open questions and suggesting points of consensus). Whilst the interviews indicated the importance of the operational researcher’s independence and neutrality, the observations suggest that the ongoing negotiation of the operational researcher’s role, and the need to establish their legitimacy in the project, did involve a political aspect (e.g. aligning their views with the meeting’s chair). Soft skills were used to perform a leadership role in encouraging the project group to participate in the goal of translating research findings from different data sources (including quantitative and qualitative data and expert opinion) into tangible recommendations for policy and practice.

**Table 4:** An account, based on non-participant observation notes, of knowledge production during project meetings and the perceived influence of the operational researcher’s role in this process

Non-participation observation of key project meetings and workshops provided insight into the practices through which knowledge was produced and ways in which OR influenced this. *Physical context: Large conference room. Two parallel screens, lots of PCs [computers] on either side of the conference room. Chairs organised roughly in rows, like an auditorium. Some participants in rows, others sitting on one side. Informal, relatively quiet, measured responses and interactions. Two hours into the meeting, a tin of biscuits appears and is circulated*. [Non-participant observation notes, November 2013] Early meetings tended to focus on the definition of key terms and categories used in the study (e.g. grouping diagnoses for the purpose of the study). Participants would ask questions, raise queries and make comments, based on their “in the moment” reading of the information being presented during meetings, by drawing on their own clinical experience or other research data, or in response to the contributions of others made during the meeting. In particular, imagined audiences for the study’s findings were periodically invoked (including parents and the media) in order to reflect critically on what the study might show and to improve its perceived quality and robustness (e.g. “might get criticised for that” and “last chance to prove how clever we are”). The to and fro of verbal contributions made during the meeting either led the group towards consensus (e.g. chair was able to state “main thing we have to do - done it, which is a miracle”) or recognition that further work to satisfy the needs of the project was still needed (e.g. one participant stated “not sure yet”, to which another responded, “we’re both not sure”). The graphic or visual aspect of the information presented by the operational researcher appears to be well received. For instance, during a lengthy, rather circular discussion, one participant refers back to the graphic that was presented earlier by the operational researcher in order to move the conversation on: “[she] gave beautiful graphic of defining index, shall we look at it again?” The operational researcher contributed to the discussions by asking clarifying questions (e.g. “what do we mean by baseline anyway?”) or suggesting points of consensus (e.g. “[are we] saying all would benefit from similar interventions?”). There appeared to be a political aspect to the ways in which the operational researcher made contributions to the study. For instance, the contributions of the operational researcher were often aligned with the chair’s views or supportive of the chair (e.g. operational researcher stated: “as [chair] said, she’s spoken with number of people on categorising”). Working closely with the chair appeared to help to legitimise the operational researcher’s role in the study and, in particular, gain favour for their approach to gathering and presenting data to inform the study’s findings. The end-of-study workshop, which was led and facilitated by the operational researcher, highlighted the importance of soft skills (or what I might term “non-academic” skills) for leading the activities of the participants present around translating the evidence collected during the study into a set of recommendations. The observations suggested that such mobilisation of knowledge demanded leadership and facilitation skills, e.g. facilitating workshops, encouraging decision-making around the data, showing participants where they might go with the findings to develop recommendations and marshalling people in different ways, e.g. by assigning action points. *The operational researcher led the meeting from the start (as chair), taking on a leadership role around ensuring the data collected during the study are translated into useful findings and tangible recommendations that can inform policy and practice. A key aspect of this was ensuring that outputs were data-driven (and not just based on participants’ own experiences). The operational researcher was able to move the participants on from discussing the data, to what it might mean for practice, and to how people in the health system might act on it. Enabling this type of discussion included pushing participants to reflect on the analysis of the data collected, e.g. in relation to the CART diagram, asking participants “are these patients groups recognisable to you?” It also seemed to require active facilitation in order to challenge at times participants’ perspectives. For example, where participants might refer to their own experience in a particular heart centre - and use this to challenge the analysis of the data presented - acknowledging and welcoming this, but also asking for broader views on the evidence presented that went beyond their personal experiences. Leadership was also needed in order to encourage participants to take forward pieces of work outside the meeting, e.g. by being firm about assigning action points*. [Reflections on end-of-study workshop, October 2014]	

## Discussion

### Findings in relation to other studies

Multidisciplinary applied health research projects often lack an explicit process or dedicated resource for integrating and tailoring the knowledge being generated to the needs of policymakers and practitioners, which can hinder its uptake [26–28]. In this study, we identified research activities associated with operational research (OR) that mediated the production of readily implementable knowledge within a research project that was successful in rapidly informing national policy. Drawing an analogy with the concept of negative space in art, where the space around and between subjects forms an interesting or relevant shape that is a significant part of the whole composition [29], the OR mediating role was shaped by an intent to perform tasks necessary to delivering translational research and yet outwith the remit of any single disciplinary strand of the research. This problem-focused rather than traditional disciplinary approach is also observed within product development, which similarly involves temporary project teams of individuals with diverse and specialised expertise collaborating within time and resource constraints towards a stated aim [30, 31]. Such teams start from a minimal shared knowledge base and so rely on explicit articulations of member’s individual knowledge and a problem-solving ethos driven by their shared goal, rather than a paradigm-driven approach based on shared narratives [30].

Solution-focused collaborative inquiry also underpins engaged scholarship, which seeks to leverage different stakeholder perspectives through meaningful interactions as a way of generating more relevant and useful knowledge [5, 17, 18]. The collaborative style of OR has previously been likened to engaged scholarship and associated with the successful co-production of knowledge for practice [21]. Our study builds on this by identifying social and material aspects of OR that empowered and enabled diverse stakeholders to interact meaningfully in generating knowledge within the context of a research project. In doing so, it contributes useful empirical insights of strategies for overcoming the potential challenges to knowledge sharing in such contexts presented by epistemological boundaries and power dynamics across different professional groups [10–14].

For example, the OR mediating role involved multiple social interactions with the eventual knowledge users to capture and incorporate their perspectives in the emerging research outputs, including facilitating a structured group workshop and holding one-to-one meetings in the build-up to the workshop. It is rare for health professionals from each part of a complex system (spanning tertiary, secondary, primary and community sectors) to meet altogether in person, so the workshop was an important opportunity for the expert group to appreciate the difficulties others were facing and consider how the system as a whole could best respond to the need to improve services. Importantly, preparatory one-to-one meetings enhanced the willingness, ability and confidence of participants to contribute effectively within the group environment of the workshop by familiarising them with the operational researcher that would facilitate it and the evidence that would be drawn on, as well as demonstrably engaging with their perspectives in advance.

Material artefacts were integral to these social interactions and an important feature of the OR mediating role. For example, the Rich Picture helped to facilitate, and was generated through, social interactions such as the one-to-one meetings, where it was used both to engage people in the research process and to capture their perspectives in a system-wide view of the problem. The CART diagram was designed and used to support the social process by which the expert group made sense of quantitative evidence and used it to inform their recommendations for improving services. The accessibility of this artefact to the less mathematically minded participants enabled discussions to be inclusive and provided a focal point. In OR, socio-material artefacts of this kind are considered to be formal models which, when produced with and used by stakeholders, can “increase their individual understandings of the problem situation of interest, help them articulate their preferences and thus enable them to appreciate the potential impact of different options, and facilitate the negotiation of courses of action that are politically feasible” [32]. Our work complements existing literature on the use of artefacts (“boundary objects”) in mobilising extant research into practice [33] by demonstrating how they can also support the sharing, integration and production of knowledge by diverse stakeholders during the conduct of multidisciplinary applied health research. In particular, it suggests that using material devices with a range of formats can help to engage stakeholders in using different forms of knowledge that may be outside their own area of expertise.

By providing an initial characterisation of OR as a mediator of knowledge production in applied health research projects, our findings build on “Behavioural OR” research to understand how behavioural aspects of OR practice support decision-making [34] and on Monks’ [19] conceptualisation of OR as an implementation science tool for structuring implementation problems, prospectively evaluating interventions and supporting strategic reconfiguration. In line with evidence that perceived fairness and transparency are prerequisites for effective interactions between stakeholders from different professions, organisations or disciplines [35, 36], our findings highlighted the importance of objectivity in the mediating role, both in terms of the person and the research processes employed. In hospital settings, the authority to broker knowledge is bound up in the person’s structural position and relationships within and between different communities [37]. Within our research project setting, a lack of strong affiliation to any one community coupled with perceived competence appeared to confer the legitimacy to constructively challenge and facilitate stakeholders and supported group creativity [38, 39]. This resonates with evidence from the co-design of public services that people have more courage to express problems with external facilitators and that the professionalism and credibility of facilitators are an important enabler to breaking through power hierarchies [40]. It also chimes with existing findings in the OR literature that the credibility of a practitioner is an important factor influencing client perceptions of quality in OR simulation studies [41]. In contrast to others in the project, who tended to be more closely affiliated with a particular community or delivering a single strand of the project, the operational researcher’s role demonstrated a commitment to participating across and joining up different communities in order to tailor the overall research findings to policy and practice.

### Strengths and limitations

The lead author of this article (SC), who conducted interviews and participant observations, also performed the OR role in the applied health project being studied. This was a study strength in that it provided rich insights into the OR approach and the project content that was useful for conducting the interviews, as well as extensive access to observations and interviewees. This enabled us to examine in detail how multidisciplinary health research was experienced and realised in practice, thus addressing an acknowledged evidence gap [9]. However, the accounts of interviewees may have been influenced by their pre-existing relationships with the interviewer (SC) and the fact that the interview was about the OR role performed by SC. In particular, participants may have been unwilling to discuss perceived weaknesses in the operational researcher’s attempt to contribute to knowledge production resulting in a bias towards perceived strengths. Acknowledging this as a potential weakness, a non-participant researcher (ST) jointly conducted some of the interviews to provide an outsider perspective and the data was critically reviewed through frequent reflective debriefs between the participant (SC) and non-participant (ST) researchers and by the wider analysis team (NJF, MU). To counterbalance the participant aspects of the study, the non-participant researcher (ST) also carried out observations at key project meetings and advisory group workshops held throughout the applied health research project. In combination, the three modes of data collection (interviews, participant observations and non-participant observations) enabled us to collect, and triangulate, data from a range of perspectives, which was a strength of the study design. In focusing on a single research project, this work is not intended to be conclusive but rather to stimulate, inform and benefit further research in other contexts.

### Implications for policymakers, researchers and clinicians

Our findings demonstrate that incorporating explicit activities, or a dedicated role, for mediating knowledge production within multidisciplinary applied health research projects warrants further consideration as a means for enhancing the implementation of research into routine healthcare. Whilst providing useful insights about the features of such a role, our research also raises important questions about who might perform it and how it might be supported. For example, could mediating activities become an explicit component of traditional disciplinary roles or are they best performed by a researcher perceived to be independent working alongside them? This need not be an operational researcher, although thematic neutrality and a problem-driven pragmatic approach may be less familiar or desirable to researchers accustomed to paradigm-driven approaches and specialisation.

Our findings describe a knowledge mediation role that encompasses a broad range of activities with both social and material aspects, including extracting and communicating insights from quantitative data, engaging people in collaborative problem solving and systems thinking, and research advocacy. Access to individual researchers experienced in the breadth of expertise required to deliver these activities is a recognised challenge [42] and would require suitable training and support. In addition, political skills would be of benefit in establishing their legitimacy and negotiating a role that involves working with, and attempting to cross, social and epistemic boundaries, suggesting the need for leadership training, including practising soft skills for leading improvement. As the OR literature acknowledges, whilst a researcher’s “background, knowledge and experience can help to bring about personal competency for mixing methods in practice” [43], a project encompassing particularly wide ranging “hard” and “soft” skills may require input from multiple researchers with complementary expertise [42]. Finally, the mediating role was found to be very time consuming, so such a role would need to be resourced sufficiently. This would require research teams to define the role in advance whilst also allowing flexibility, and research funders to recognise it as legitimate costed activity.

## Conclusions

The potential value of producing readily implementable knowledge in multidisciplinary applied health research projects is recognised but challenging to realise [9, 44]. Our study contributes useful empirical insights about effective knowledge production in such settings and raises important questions about how to incorporate and resource knowledge mediation activities within projects. Empirical studies in other contexts will be important to testing and theoretically developing this work further.

## Additional files

Additional file 1: The Rich Picture developed as part of the operational research approach. This Rich Picture (a device used in soft systems methodology) was developed to explore the key features of services following infant cardiac surgery, perceived problems and possible improvements (reproduced from [45]). (PDF 2093 kb)

Additional file 2: The CART diagram developed as part of the operational research approach. This visual representation of data analysis (the CART diagram) was created to inform a decision process around prioritisation of service improvements (reproduced from [45]). (PDF 247 kb)

## Abbreviations

CART: Classification and regression tree analysis
MU: Martin Utley (author)
NHS: National Health Service
NJF: Naomi J Fulop (author)
OR: Operational Research
PC: Personal computer
SC: Sonya Crowe (author)
SSM: Soft systems methodology
ST: Simon Turner (author)
UK: United Kingdom
