# Supplementary figures and images for: Improving the production of applied health research findings: insights from a qualitative study of operational research
Source: Implement Sci. 2017 Sep 8;12:112. doi: 10.1186/s13012-017-0643-3 (PMC5591553; doi:10.1186/s13012-017-0643-3)

COMMUNICATION!

"overseeing eye"

"touch point"  
"stock broker"

TRUST

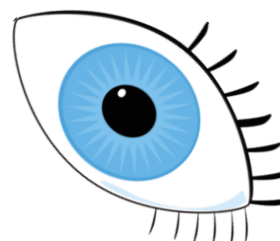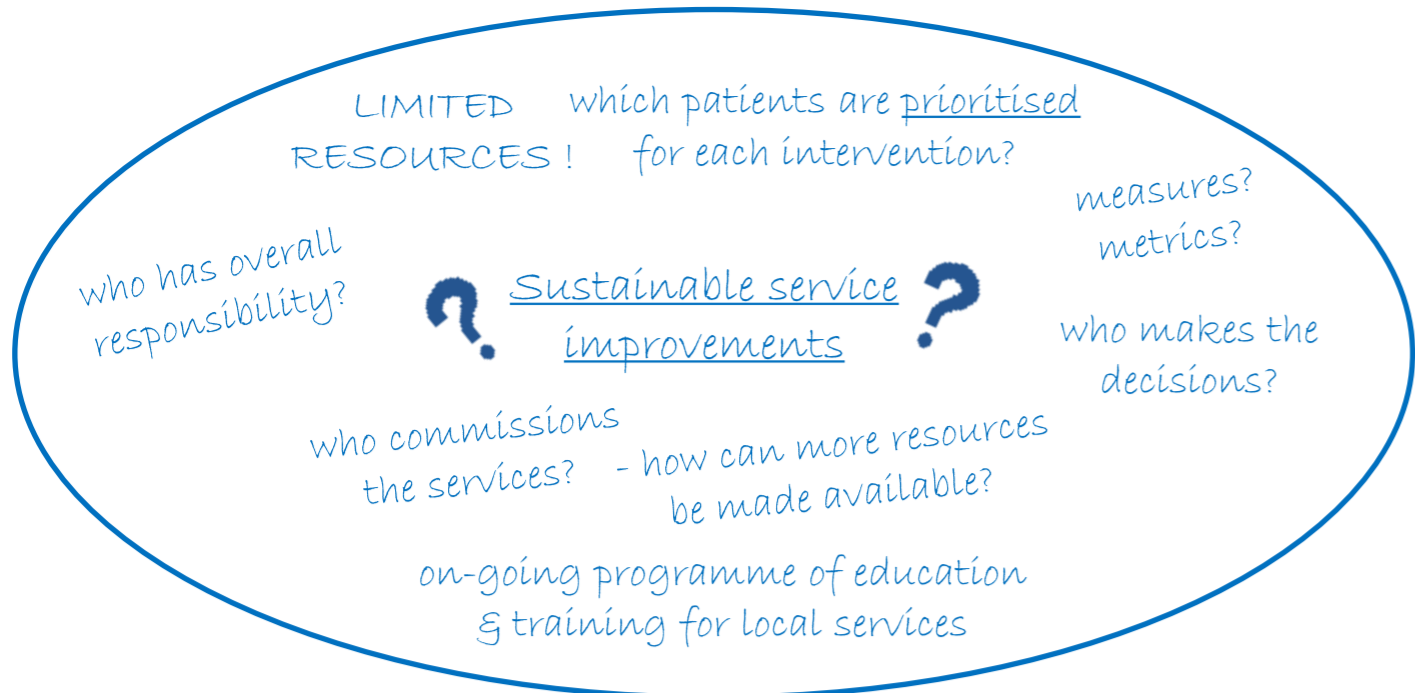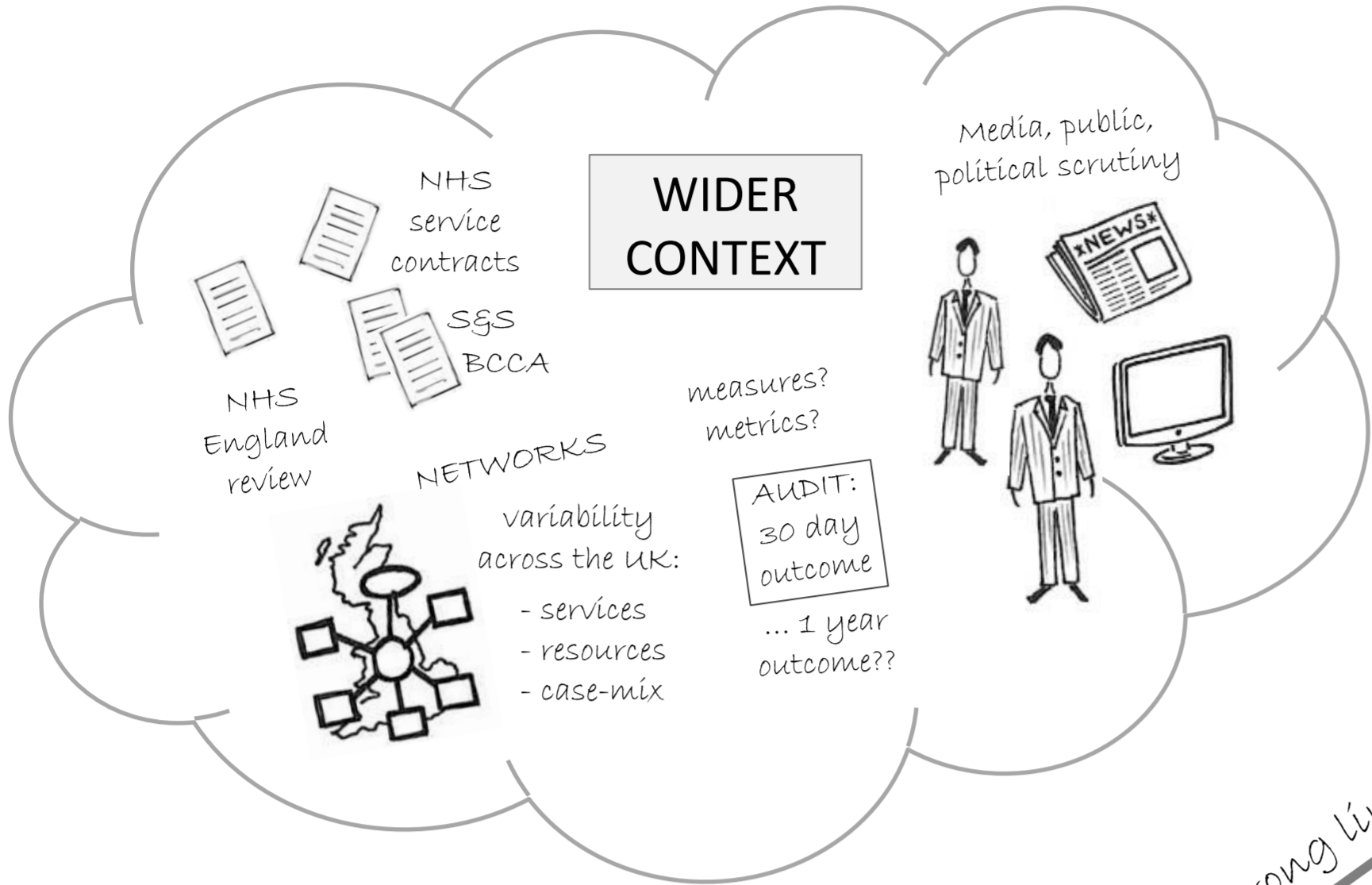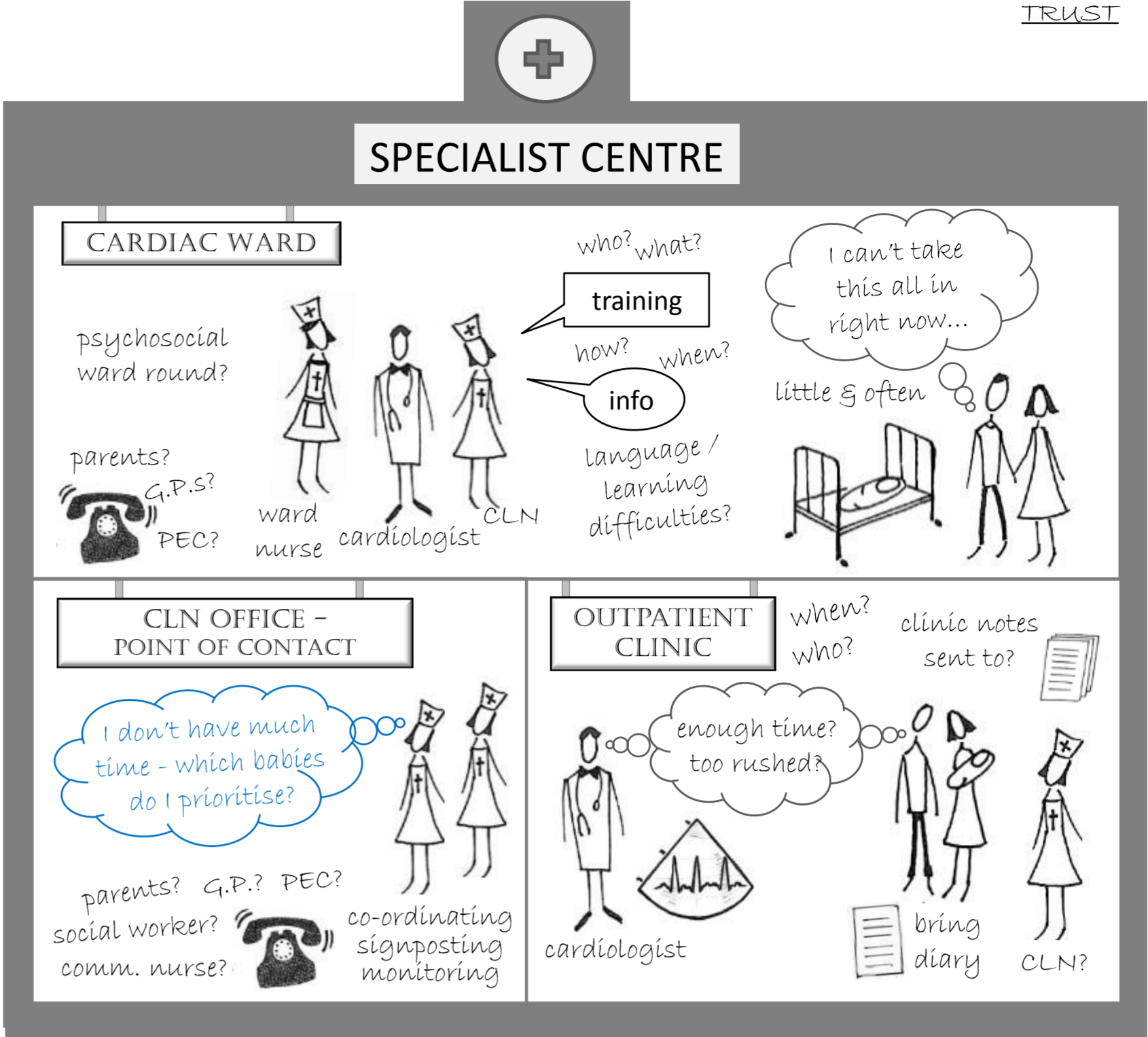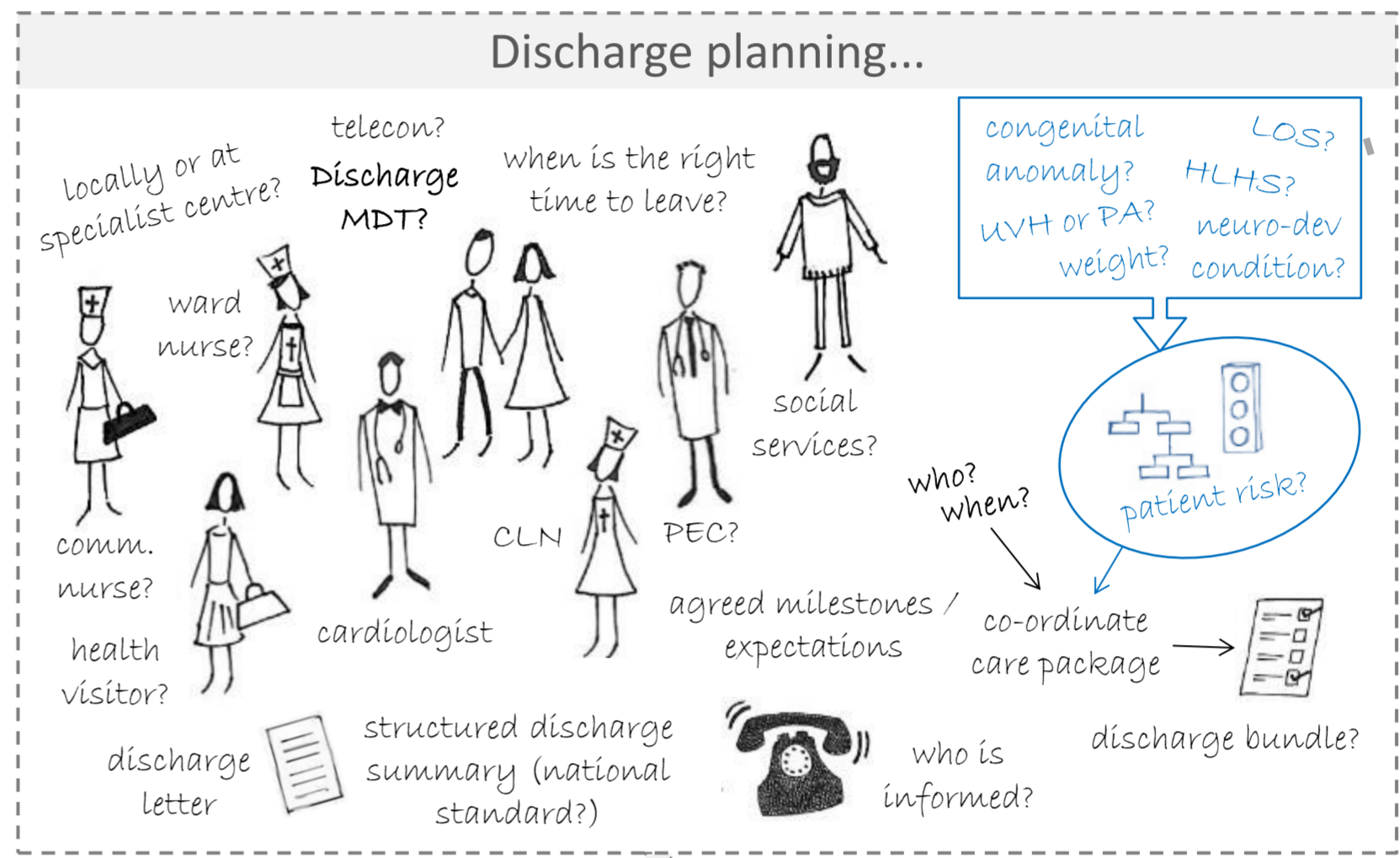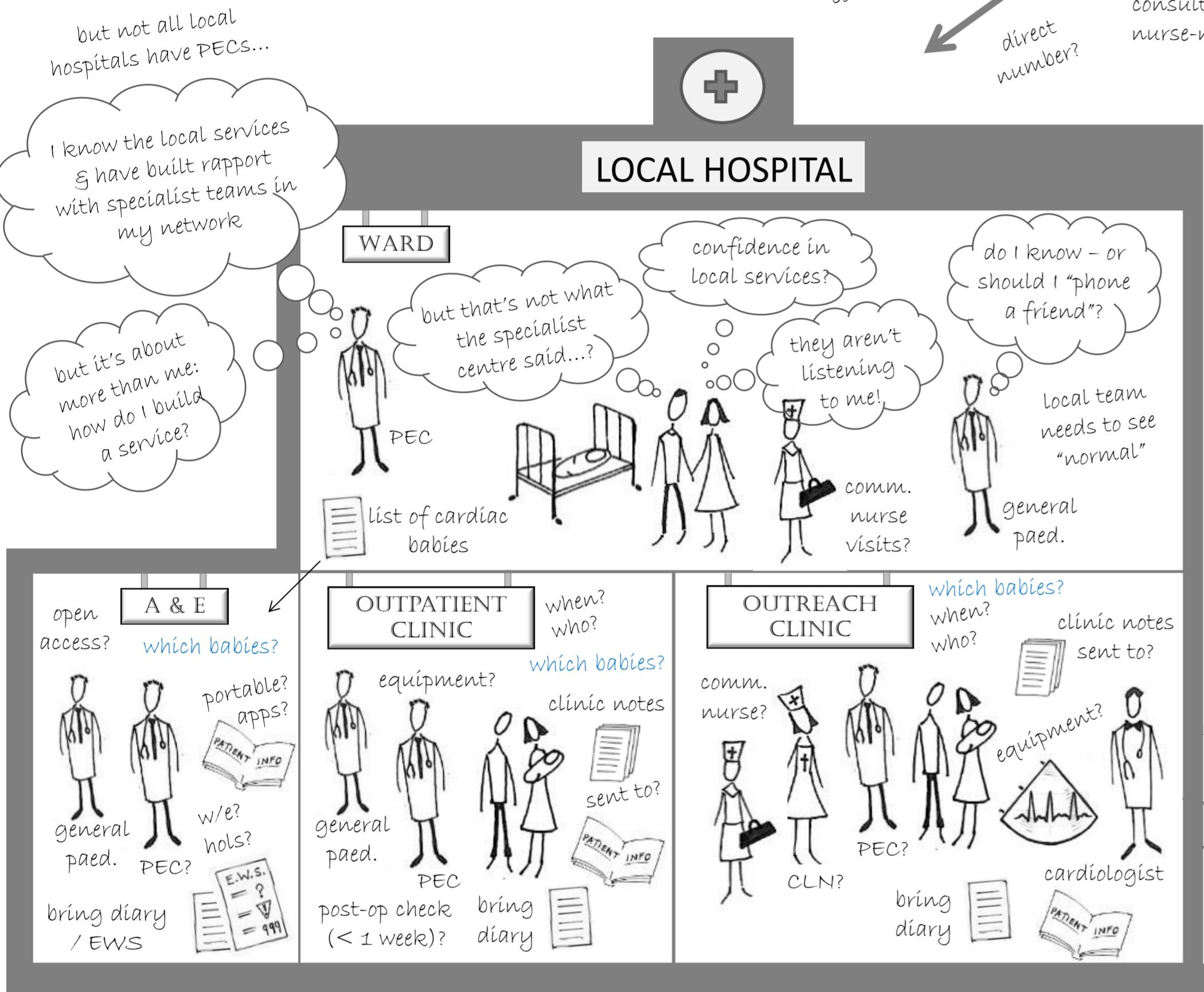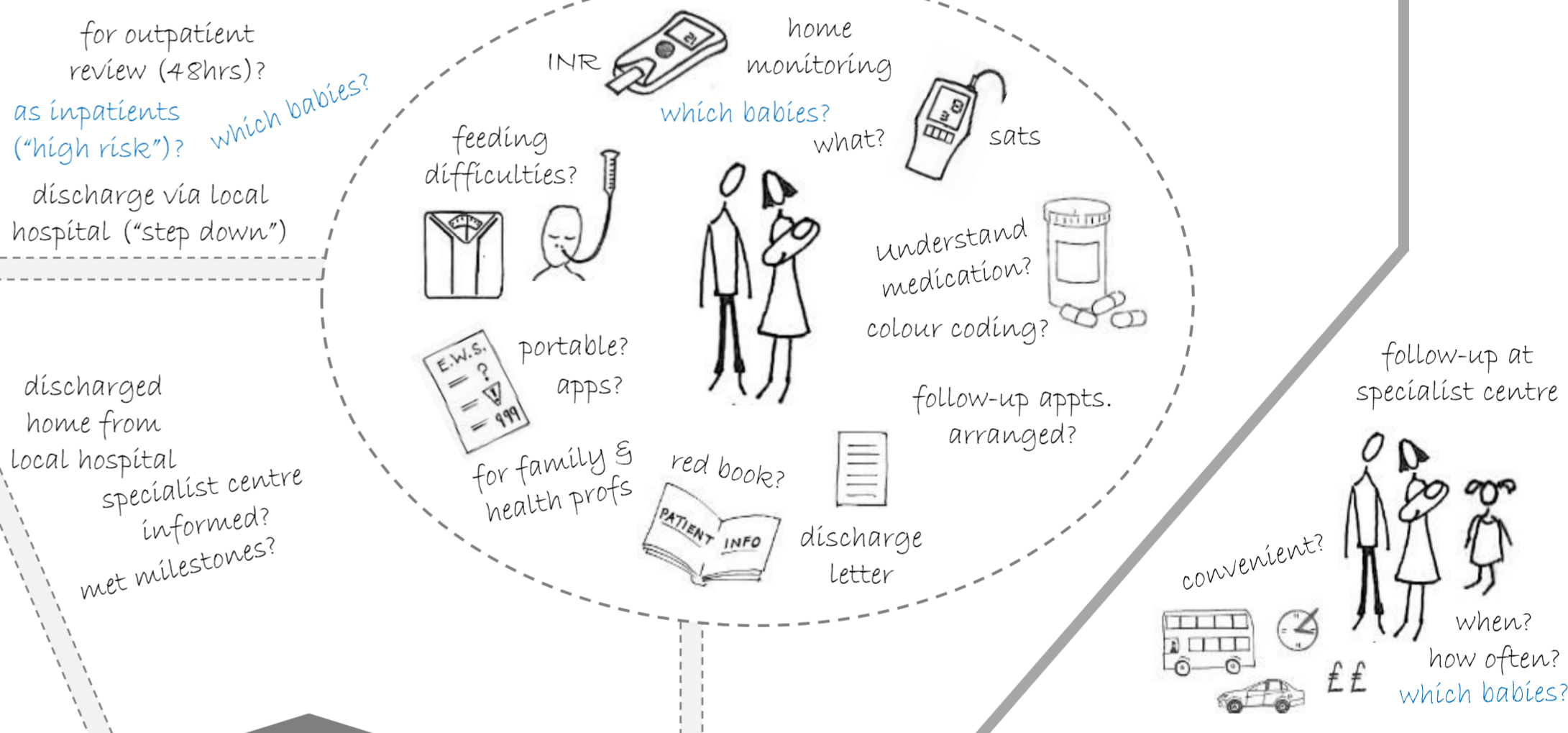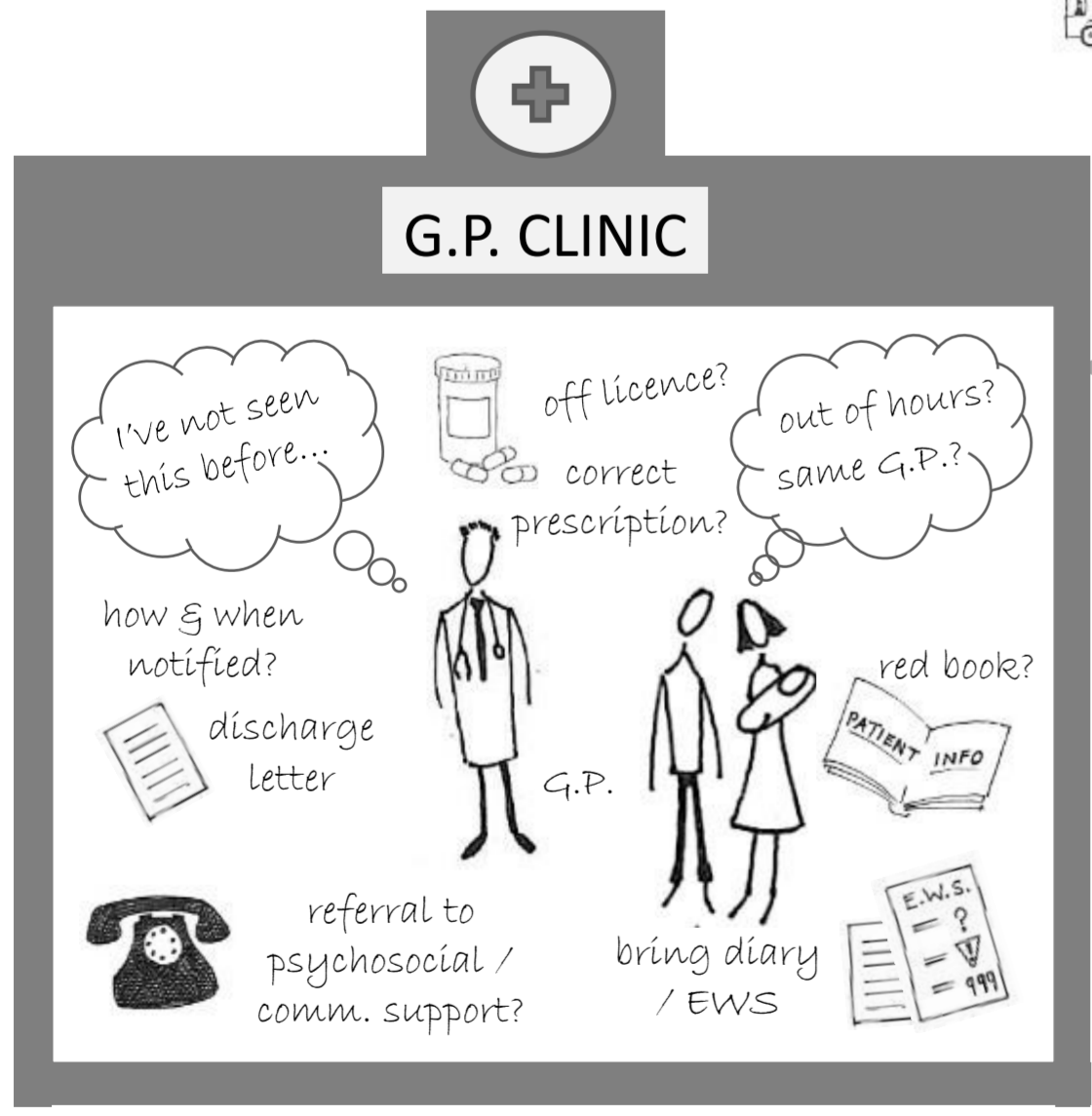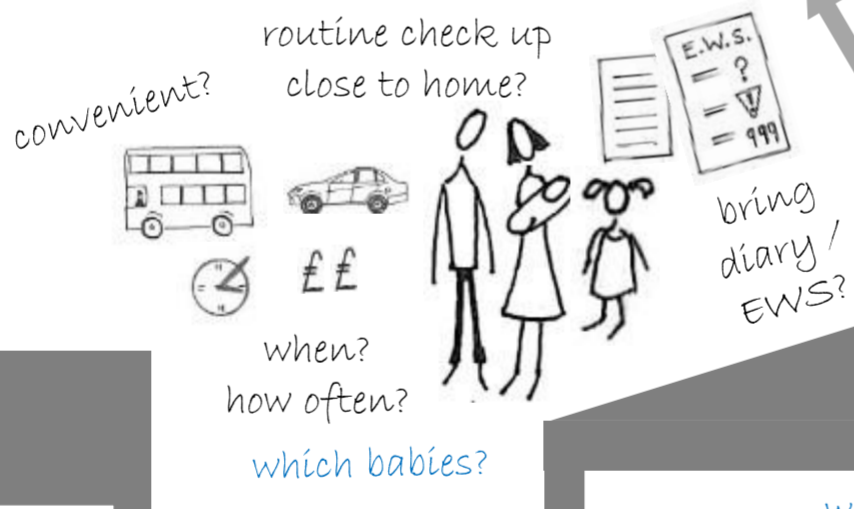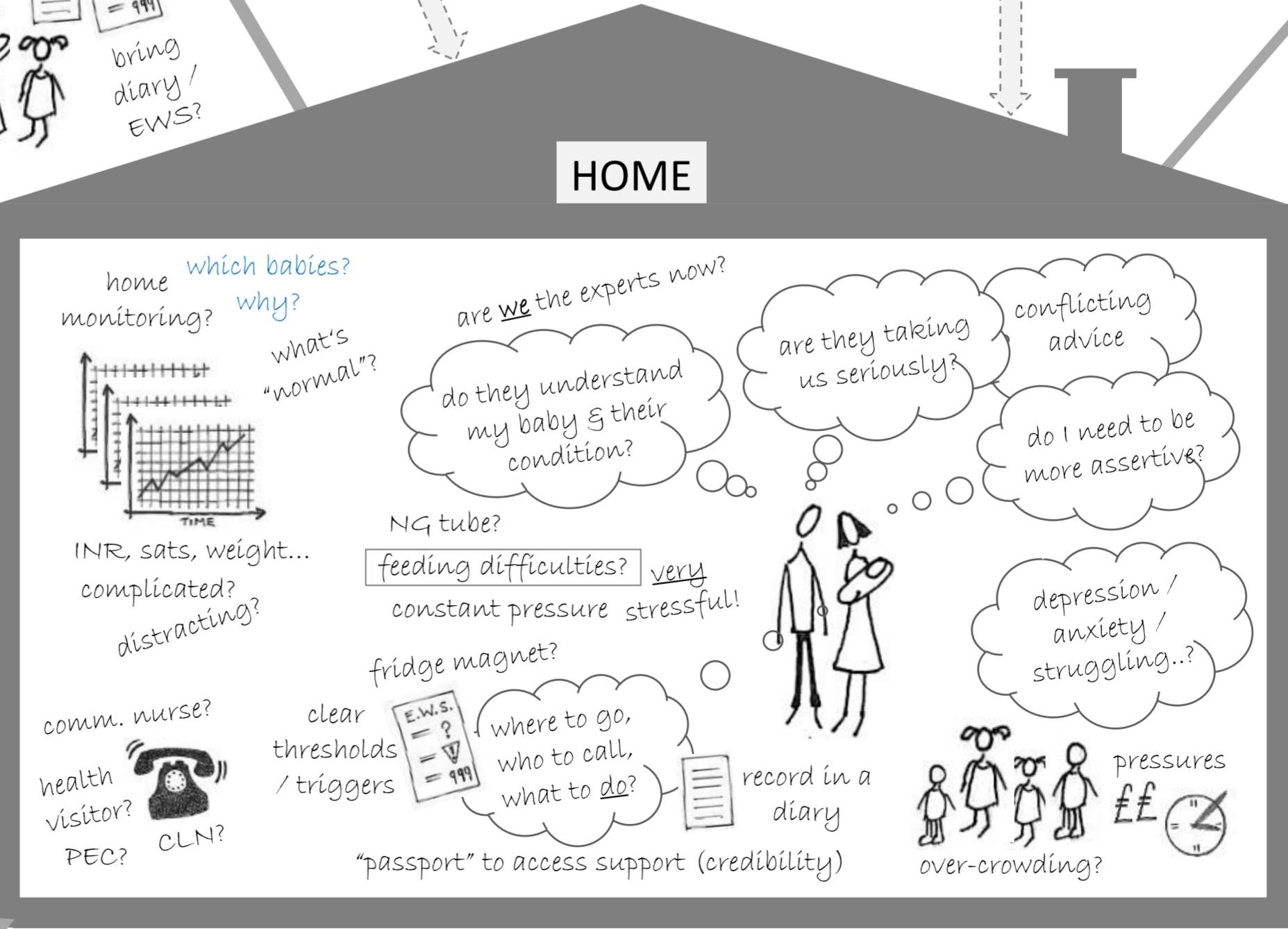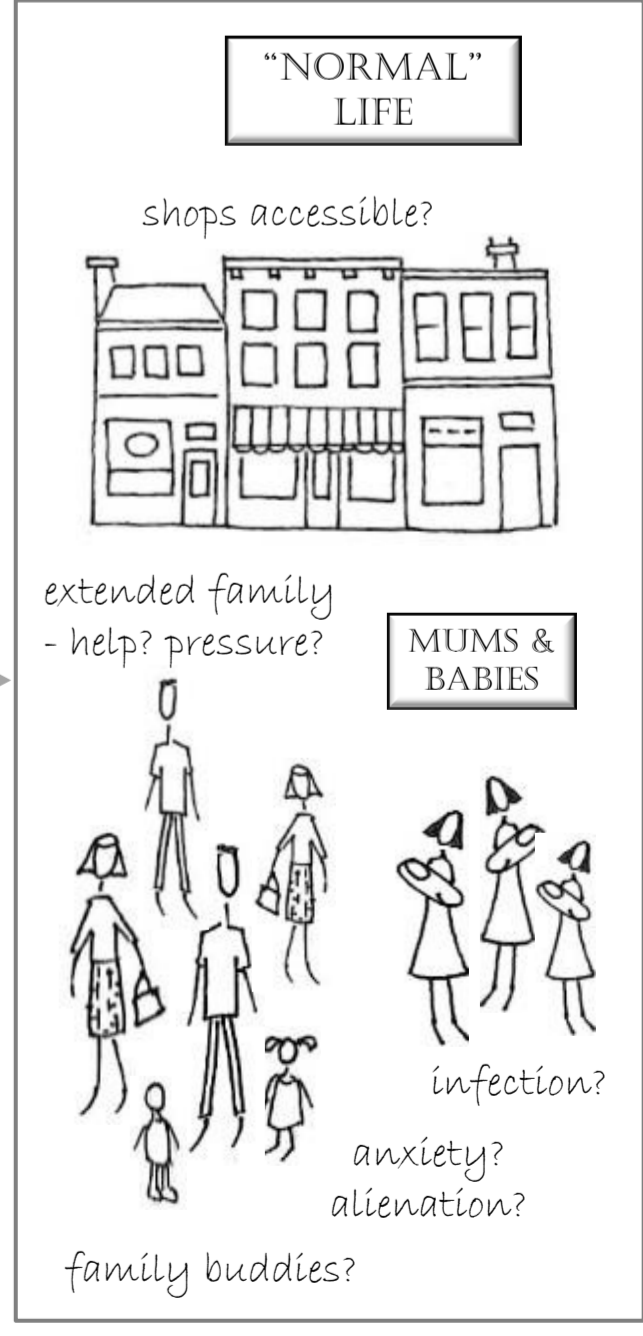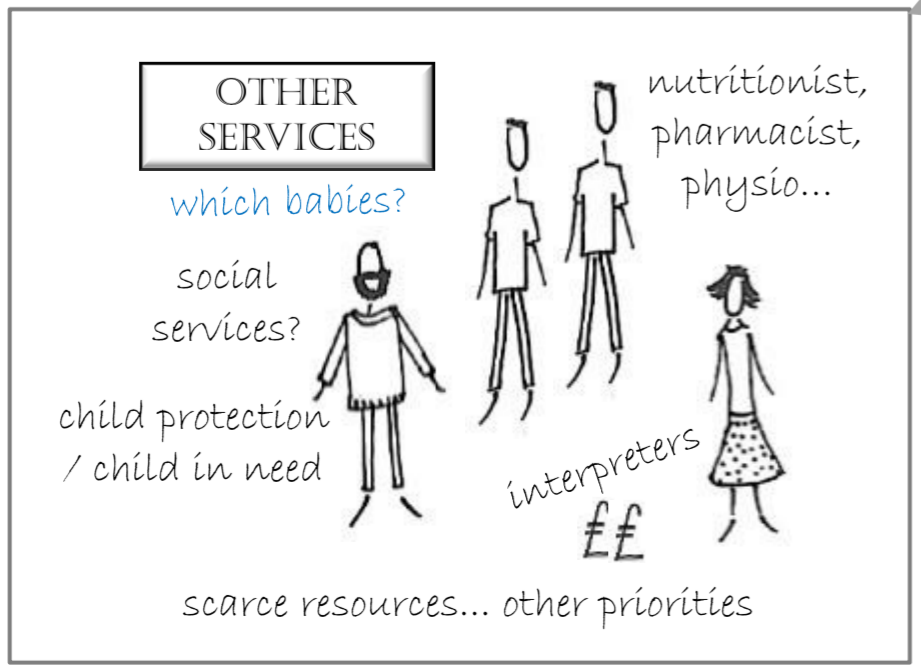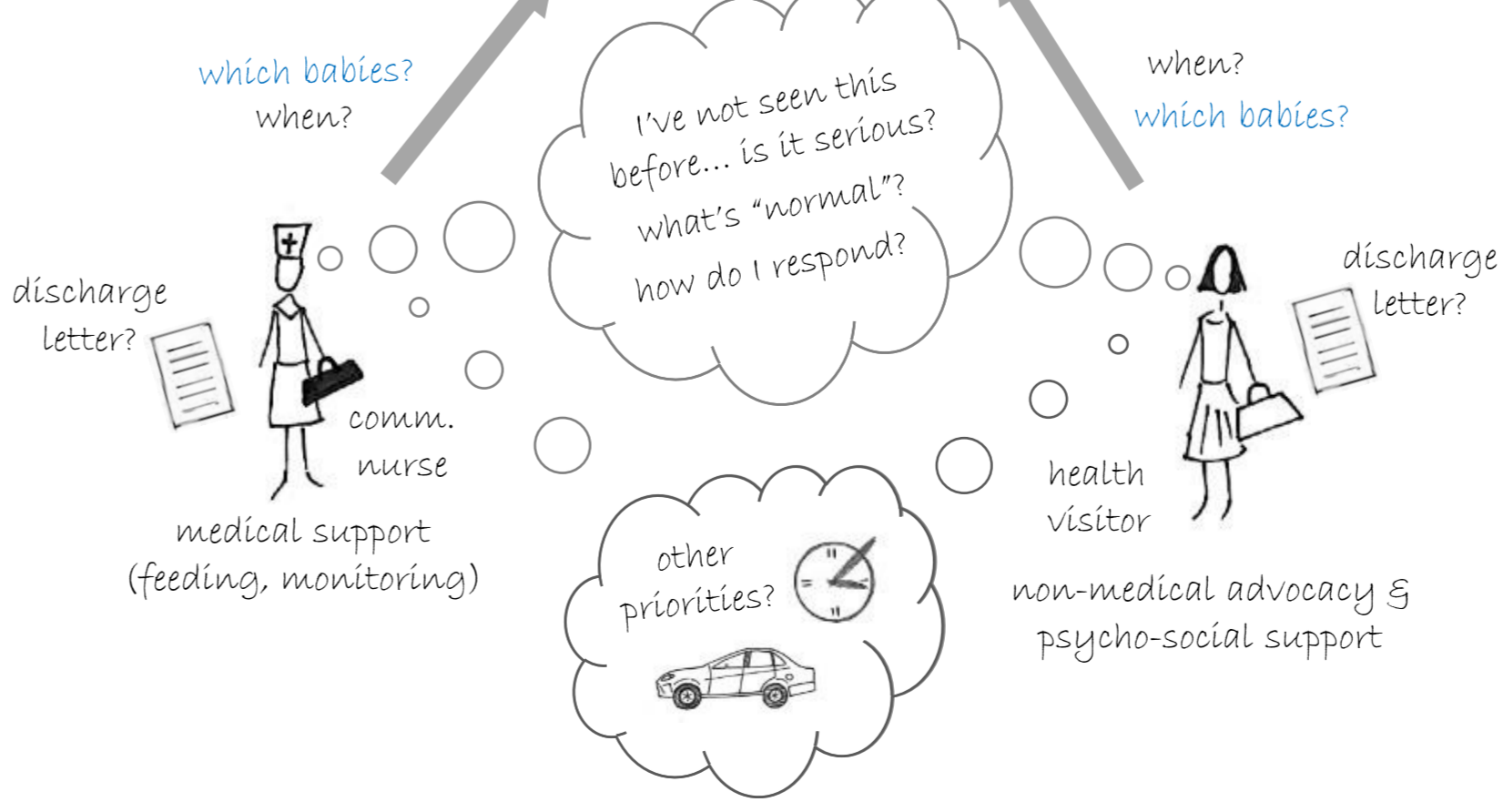

Supplement: Supplementary file 1 — The Rich Picture developed as part of the operational research approach. This Rich Picture (a device used in soft systems methodology) was developed to explore the key features of services following infant cardiac surgery, perceived problems and possible improvements (reproduced from [45]). (PDF 2093 kb) [file 13012_2017_643_MOESM1_ESM.pdf]

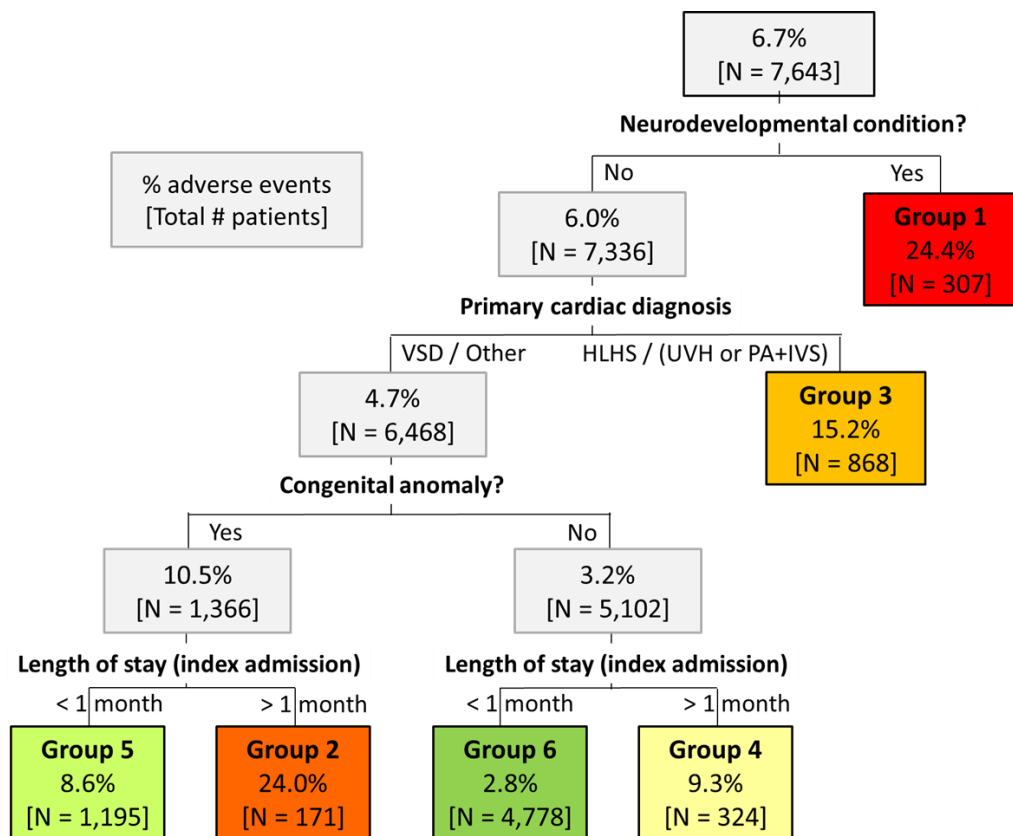

Cumulative proportion of adverse events

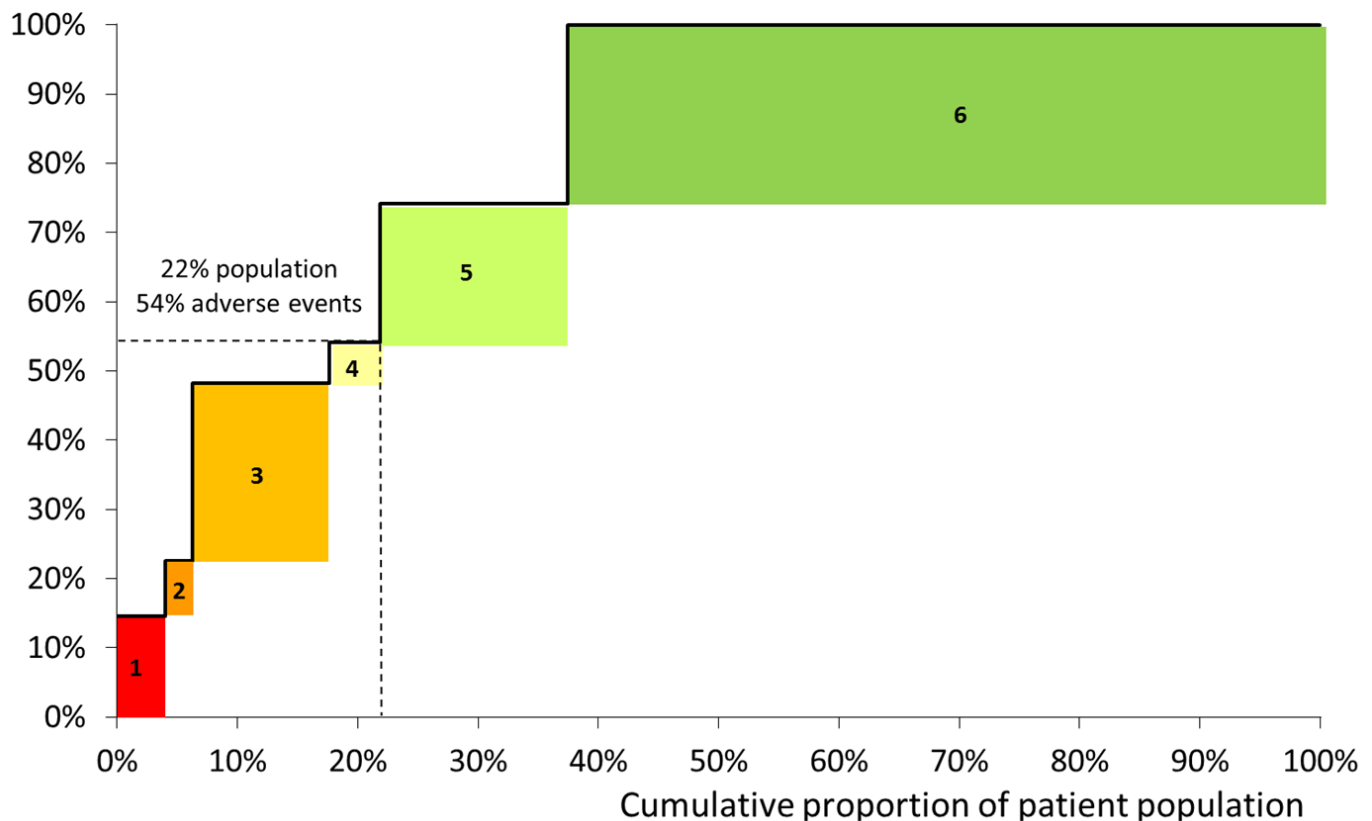

Supplement: Supplementary file 2 — The CART diagram developed as part of the operational research approach. This visual representation of data analysis (the CART diagram) was created to inform a decision process around prioritisation of service improvements (reproduced from [45]). (PDF 247 kb) [file 13012_2017_643_MOESM2_ESM.pdf]
